# Diversity of Dengue Virus Serotype in Endemic Region of South Sulawesi Province

**DOI:** 10.1155/2018/9682784

**Published:** 2018-04-23

**Authors:** Muh. Taslim, A. A. Arsunan, Hasanuddin Ishak, Sudirman Nasir, Andi Nilawati Usman

**Affiliations:** ^1^Doctoral Program, Faculty of Medicine, Hasanuddin University, Makassar, Indonesia; ^2^Syech Yusuf Hospital, Gowa Health Office, South Sulawesi, Indonesia; ^3^Department of Epidemiology, Faculty of Public Health, Hasanuddin University, Makassar, Indonesia; ^4^Department of Environmental Health, Faculty of Public Health, Hasanuddin University, Makassar, Indonesia; ^5^Department of Health Promotion, Faculty of Public Health, Hasanuddin University, Makassar, Indonesia; ^6^Department of Public Health, Mandala Waluya College, Kendari, Indonesia; ^7^Department of Midwifery, Postgraduate School, Hasanuddin University, Makassar, Indonesia

## Abstract

The objective of this research was to investigate serotype diversity pattern of dengue hemorrhagic fever virus by using real-time-polymerase chain reaction (RT-PCR) method. It was an explorative laboratory research in endemic dengue fever area in South Sulawesi Province, Indonesia, that is, Makassar municipality and Maros and Gowa region. Serological examination was carried out using real-time-polymerase chain reaction (RT-PCR) method to determine the serotype of dengue virus. The data showed that, of 30 patients, 20 patients (66.67%) were from Makassar municipality: 10 patients (33.33%) from Gowa region and 10 patients (33.33%) from Maros region. The serotypes found were DENV-2 and DENV-4 and no DENV-1 and DENV-3 serotypes were found. Makassar municipality and Gowa region have higher infection with serotype DENV-2, that is, 40% of cases compared with Maros, which is 20.0%. Statistical test results showed no significant differences between the three endemic areas. Maros region has the highest infection with serotype DENV-4, that is, 40% of cases compared with Makassar municipality (5.0%) and Gowa region (0%). Statistical test results showed significant differences between the three endemic areas. This result revealed that serotypes obtained in endemic areas of dengue fever in South Sulawesi are DENV-2 and DENV-4 and not serotypes DENV-1 and DENV-3. Makassar municipality has DENV-2 and DENV-4 serotype, infection dominated by DENV-2, while Maros region also has DENV-2 and DENV-4, but DENV-4 is the dominant serotype. Gowa municipality only has DENV-2 serotype infection.

## 1. Introduction

Dengue hemorrhagic fever (DHF) is an infectious disease caused by dengue virus spread by* Aedes aegypti* mosquito as the main vector. It is still a public health problem; epidemics and endemics continue to occur especially in the tropics and subtropics area such as Indonesia. Over the last 45 years despite various efforts being made, it seems the incidence of dengue fever still continues to increase and Indonesia is categorized as high endemic country [1, 2].

Dengue virus belongs to arthropod-borne virus group, family Flaviviridae, and genus* Flavivirus*. Family Flaviviridae is associated with severe disease and high mortality in animals or humans [3, 4]. Dengue virus has four serotypes, namely, DENV-1, DENV-2, DENV-3, and DENV-4. Serotype of dengue virus has correlation with severity of dengue fever due to host immune response. Immunity will form when a person is infected with one type of serotype but does not apply when a secondary infection develops with another serotype; this can actually aggravate the disease until it is life-threatening [5].

Detection and mapping of serotypes in an area can be a strategy to monitor transmission of dengue viruses and investigate outbreak especially preventing severe outcomes in the event of outbreak or in the case of other handling [6]. A meta-analysis study found that in Southeast Asia (SEA) severe cases of dengue fever were dominated by the DENV-3 serotype [7].

Some of the areas where dengue survey by the research has been conducted show different results; for example, a cohort survey in the Indonesian capital, Jakarta, in 2009-2010, shows that only DENV-4 dominates the infection but a case study conducted in 2013 showed that severe cases of dengue were caused by infection with serotype DENV-3 [8]. A cross-sectional study conducted in Bali in 2015 found that DENV-3 was predominant [9]. Makassar, capital of South Sulawesi Province, based on dengue fever surveillance year 2007–2010 conducted on a study showed that DENV-1 is the most dominant following DENV-2, DENV-3, and DENV-4 [10]. This data indicates that predominant serotypes in a particular area and time vary and also the severity of dengue fever is closely related to serotype; this study attempts to record dominant serotypes. The purpose of this study is to examine the diversity pattern of serotype virus disease dengue hemorrhagic fever from endemic area in South Sulawesi, specifically Makassar, Maros, and Gowa region, using real-time-polymerase chain reaction (RT-PCR) method.

## 2. Material and Methods

### 2.1. Study Design and Area

This study was an explorative laboratory type research by examining serotype dengue virus in DHF patients in dengue endemic areas: Gowa, Makassar, and Maros regions.

### 2.2. Samples of Study

Samples of study were DHF patients that are treated in hospitals in endemic areas in Makassar as many as 20 patients, in Gowa as many as 10 patients, and in Maros as many as 10 patients. Sampling method used purposive sampling with inclusion criteria: having a rectal temperature higher than 38.0°C for a period of fewer than 48 hours, the presence of informed consent from patients willing to participate in the study, the clinical suspicion that the patient has dengue fever, and and NS1 antigen test being positive.

### 2.3. Ethical Approval

The study was approved by ethics committee of medical faculty of Hasanuddin University

### 2.4. Serotyping Examination

Laboratory examination was conducted at biomolecular laboratory of medical faculty of Hasanuddin University using RT-PCR technique for dengue virus serotype examination.

#### 2.4.1. RNA Extraction

Extraction of RNA virus was performed using QIAamp Viral RNA Mini Kit (Qiagen), protocol based on manufactory instruction.

#### 2.4.2. RT-PCR Detection and Serotyping

The probes are labeled with FAM from Macrogen Korea. Serotypes of DENV-1, DENV-2, DENV-3, and DENV-4 viruses were detected using real-time PCR assay in 25 *μ*L PCR reaction mix containing 5 *μ*L RNA template, TaqMan® Fast Virus 1-Step Master Mix (Applied Biosystems®), UltraPure™ DNase/RNase-Free Distilled Water (Invitrogen™), and a primary 0.9 and a 0.2 *μ*M probe, respectively. The probe is labeled with a dye and a nonfluorescent quencher FAM reporter. Primers and probes were obtained from Macrogen, Seoul, Korea.

Amplification and detection were carried out using StepOnePlus real-time PCR system (Applied Biosystems). The cycle and temperature used were reverse-transcription at 50°C for 5 minutes, inactivation at 95°C for 20 seconds, and then 45 fluorescence detection cycles at 95°C for 3 minutes and annealing at 60°C for 30 seconds. The baseline and threshold baseline have been set automatically by using the RT-PCR tool set in StepOne Software v2.2.2 (Applied Biosystems).

The sample is said to be positive if the amplification target is recorded in 40 cycles. CDC DENV-1–4 Real-Time RT-PCR Assay used singleplex reaction adjusted to the instructions of the reagent (Centers for Disease Control and Prevention) in 25 *μ*L volumes using SuperScript® III Platinum® One-Step qRT-PCR Kit (Invitrogen) (primary design, 2016).

Real-time PCR amplification and dengue virus detection were carried out using Biorad Real-Time PCR (USA) tool. The data is made as per the company's instructions and briefly the threshold arranged in the exponential phase of PCR can be seen linearly. The specimen is said to be either DENV-1, DENV-2, DENV-3, or DENV-4 if the cross-line amplification curve is in 37 cycles (Cq < 37).

### 2.5. Statistical Analysis

Data was displayed in table form with frequency and percentage and statistical analysis using chi-square. The probability value is considered significant when it is less than 0.05.

## 3. Result

The data showed that, of 30 patients, 20 patients (50.0%) were from Makassar municipality: 10 patients (25.0%) from Gowa region and 10 patients (25.0%) from Maros region. The serotypes of dengue virus in endemic areas of Makassar, Gowa, and Maros were DENV-2 and DENV-4 and no DENV-1 and DENV-3 serotypes were found (Table 1).

Serotype detection with PCR showed that, in Makassar municipality, serotype DENV-2 frequency was higher than serotype DENV-4; it is different with Maros region which was dominated by DENV-4 compared to DENV-2 while in Gowa region all infections detected were DENV-2 and no other types were found (Table 1).

Makassar municipality and Gowa region have higher infection with serotype DENV-2, that is, 40% of cases compared with Maros, which is 35.0%. Statistical test results showed no significant differences between the three endemic areas (Table 2).

Maros region has the highest infection with serotype DENV-4, that is, 40% of cases, compared with Makassar municipality (50.5%) and Gowa region (0%). Statistical test results showed significant differences between the three endemic areas (Table 3).

## 4. Discussion

This study revealed that serotype diversity among the three dengue fever endemic areas in South Sulawesi did occur. The serotypes of dengue virus in endemic areas of Makassar, Gowa, and Maros were DENV-2 and DENV-4 and no DENV-1 and DENV-3 serotypes were found. Serotype DENV-2 frequency was higher than serotype DENV-4; it is different with Maros region which was dominated by DENV-4 compared to DENV-2 while in Gowa region all infections detected were DENV-2 and no other types were found. Makassar and Maros have diverse serotypes, that is, DENV-2 and DEN-4, while Gowa only has one type, that is, DENV-2.

There are variations across time and between geographical locations of dominant serotype in dengue fever infection. The dominant serotype in Indonesia is DENV-3, but in some studies as those done in Bandung in 2000–2002 it was found that DENV-2 is the most common serotype found. A cohort study in Jakarta in 2009-2010 actually shows that DENV-4 is the most dominant and a surveillance study in Makassar in 2007–2010 found that DENV-1 was the dominant serotype for infection [9, 10].

Several studies on serotypes and the severity of dengue fever have been published. A study in Brazil showed that DENV-2 had more severe clinical manifestations than DENV-1 and DENV-4; this is similar to data that is the result of studies in Thailand; DENV-2 is associated with severe dengue infection while other studies in Singapore have shown that DENV-1 is more indicative of severe clinical manifestations when compared with DENV-2. Clinically, patients infected with DENV-1 tend to have red eyes while DENV-2 infection results in joint pain and low platelet count [6, 11, 12]. The severity of the disease is also seen in Indonesia; in Sumatra it was found that serotype DENV-1 infection was associated with a nonsevere dengue incidence [13].

Information on the types of serotypes present in a region can be used to determine the potential for severe conditions of febrile illness in an area as well as for the development and application of appropriate vaccines. Prevention and treatment in outbreaks are also expected to be easier with the knowledge of this dengue virus serotype. Current vaccine development has been done in various ways including live attenuated, inactivated, recombinant subunit, DNA, and viral vectored vaccines [14, 15]. However, vaccine development by combination to protect all types of DENV still needs evaluation; it will be better to use serotype mapping to develop vaccine [16–18].

This study needs to be continued with a more significant number of samples and phylogenetic analyses and their correlations with disease severity and clinical manifestations.

## 5. Conclusion

Serotypes obtained in endemic areas of dengue fever in South Sulawesi are DENV-2 and DENV-4 and not serotypes DENV-1 and DENV-3. Makassar municipality has DENV-2 and DENV-4 serotypes, infection dominated by DENV-2, while Maros region also has DENV-2 and DENV-4 but DENV-4 is the dominant infection. Gowa municipality only has DENV-2 serotype infection.

## Figures and Tables

**Table 1:** Distribution of DENV virus serotype in Makassar, Gowa, and Maros.

Serotype			Region					
	Makassar		Gowa		Maros			
	(*n* = 20)		(*n* = 10)%		(*n* = 10)		Total	
							Positive	Negative
	*n* (%)		*n* (%)		*n* (%)			
	Positive	Negative	Positive	Negative	Positive	Negative		
DENV-1	0 (0)	20 (100)	0 (0)	10 (100)	0 (0)	10 (100)	0 (0)	40 (100)
DENV-2	8 (40)	12 (60)	4 (40)	6 (60)	2 (20)	8 (80)	14 (35)	26 (65)
DENV-3	0 (0)	20 (100)	0 (0)	10 (100)	0 (0)	10 (100)	0 (0)	40 (100)
DENV-4	1 (5)	19 (95)	0 (0%)	10 (100)	4 (40)	6 (60)	5 (12,5)	35 (87,5)

**Table 2:** Comparison of DENV-2 serotypes in endemic areas of dengue fever in Makassar, Gowa, and Maros.

DENV-2	Municipality/region								*p*
	Makassar		Gowa		Maros		Total		
							*n*	%	
	*n*	%	*n*	%	*n*	%			
Positive	8	40,0	4	40,0	2	20,0	14	35,0	0,517
Negative	12	60,0	6	60,0	8	80,0	26	65,0	
Total	20	100,0	10	100,0	10	100,0	40	100,0	

**Table 3:** Differences in DENV-4 diversity in endemic areas of dengue fever in Makassar, Gowa, and Maros.

DENV-4	Municipality/region								*p*
	Makassar		Gowa		Maros		Total		
							*n*	%	
	*n*	%	*n*	%	*n*	%			
Positive	1	5,0	0	0,0	4	40,0	5	12,5	0,009
Negative	19	95,0	10	100,0	6	60,0	35	87,5	
Total	20	100,0	10	100,0	10	100,0	40	100,0	
